# Active control on topological immunity of elastic wave metamaterials

**DOI:** 10.1038/s41598-020-66269-2

**Published:** 2020-06-10

**Authors:** Guan-Hua Li, Tian-Xue Ma, Yi-Ze Wang, Yue-Sheng Wang

**Affiliations:** 1 Institute of Engineering Mechanics, Beijing Jiaotong University, Beijing, 100044 China; 2 Department of Civil Engineering, University of Siegen, Siegen, D-57068 Germany; 3 Department of Mechanics, Tianjin University, Tianjin, 300350 China

**Keywords:** Civil engineering, Mechanical engineering

## Abstract

The topology concept in the condensed physics and acoustics is introduced into the elastic wave metamaterial plate, which can show the topological property of the flexural wave. The elastic wave metamaterial plate consists of the hexagonal array which is connected by the piezoelectric shunting circuits. The Dirac point is found by adjusting the size of the unit cell and numerical simulations are illustrated to show the topological immunity. Then the closing and breaking of the Dirac point can be generated by the negative capacitance circuits. These investigations denote that the topological immunity can be achieved for flexural wave in mechanical metamaterial plate. The experiments with the active control action are finally carried out to support the numerical design.

## Introduction

Phononic crystals and elastic wave metamaterials are artificial structures which are arranged periodically and have received lots of attention^1–8^. These new kinds of structures have many extraordinary properties, e.g. the wave band gaps^9–12^, negative refraction^13,14^, acoustic/elastic wave cloaks^15–17^, etc. These interesting behaviors can be used to tune the wave propagation properties and have a wide variety of potential engineering applications. Acoustic couplers^18^, sensors^19^ and waveguides^20^ are several typical applications of phononic crystals and elastic wave metamaterials.

In recent years, a lot of attention has been focused on the extraordinary transmission phenomena of acoustic/elastic waves near the Dirac point. The periodic structures with the Dirac point can present many interesting behaviors, e.g. carrier mobility^21^, zero refractive index^22–25^, Hall effect^26^ and topological edge states^27–30^, etc. It should be mentioned that in the photonic crystal, the spatial phase can be reconstructed by the degenerate Bloch modes at the Dirac point^31^. Moreover, the topological phase can also transform because of the band inversion^32^.

On the other hand, some investigations have been reported on the phononic crystals and elastic wave metamaterials with the active control action^33–37^. To present the controllable flexural waves with the broadband characteristics by the active control, an experiment using the shunted piezoelectric patches was reported^38^. Furthermore, another experiment was performed to show the tunable waveguide of the phononic plate^39^. Although the active control action has been applied on the waveguide structures, its tunable effects on the topological properties of elastic wave metamaterials have not been considered. In this work, we propose a tunable topological state of the flexural wave with the active control. Based on the electrical control action, the Dirac point and its corresponding topological immunity can be achieved.

## Methods

**Figure 1:**
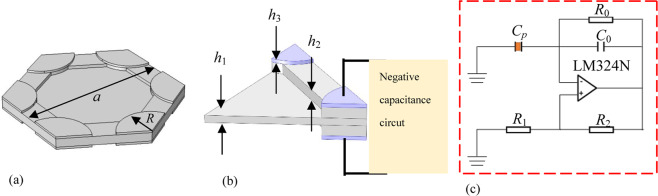
(**a**) A unit cell bonded by piezoelectric patches, (**b**) the piezoelectric patches attached by the negative capacitance circuits and (**c**) the negative capacitance circuit.

In this work, we focus on elastic wave metamaterials with double Dirac points locating at the center of the Brillouin zone. A hexagonal unit cell in the elastic wave metamaterial plate is showed in Fig. 1(a). The cell is made of the resin and its six corners are attached with the piezoelectric material P-4 on both sides. The parameters are illustrated in Table 1, where *a* is the lattice constant, *R* is the radius of the piezoelectric patch, *h*_1_–*h*_3_ are the thicknesses of the middle layer, groove and piezoelectric sheet, respectively. Each piezoelectric patch is connected by a negative capacitance circuit to behave as the active control load in Fig. 1(b). As shown in Fig. 1(c), the negative capacitance circuit is used in the active control system to change the elastic modules, in which the capacitance *C*_*p*_, compensation resistance *R*_0_, operational amplifier, fixed resistances *R*_1_ and sliding rheostat *R*_2_ are considered. The derivation of the negative capacitance circuit is presented in Supplementary Material. Both the dispersion curves and propagation properties are calculated by the finite element software COMSOL. Our attention is focused on the topological immunity of the elastic wave in which the energy bands of the flexural modes are presented.

**Table 1:** The parameters of the unit cell.

*a* (mm)	*h*_1_ (mm)	*h*_2_ (mm)	*h*_3_ (mm)	*h*_4_ (mm)
$40\sqrt{3}$	1	1.554	1	15

## Results and discussions

**Figure 2:**
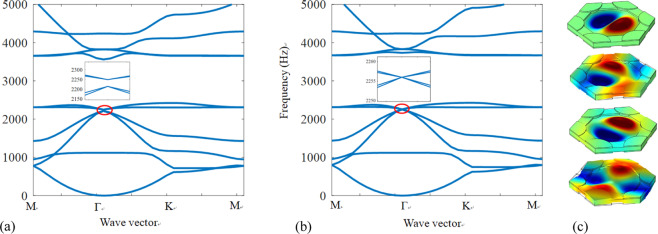
(**a**) Band gap, (**b**) the Dirac point induced by the electric circuit and (**c**) its displacement fields of the four degenerate states at the Dirac point.

When the negative capacitance circuits are not connected, the band structure calculated by the finite element method is shown in Fig. 2(a) and we can see that the band gap width is about 35 Hz. In order to generate the Dirac point, the negative capacitance circuits are connected and the parameter of the resistance *α* = 0.9 in Eq.(A.4) is applied. The band structure with the Dirac point is shown in Fig. 2(b), in which a quadruple degeneracy at 2256 Hz can be observed. The displacement field distributions for the four degenerate states at the Dirac point are presented in Fig. 2(c).

An interesting behavior is the extraordinary transmission at the Dirac cone. In this work, it is designed by the configuration and electrical tuning to show the immune property of the elastic wave to the object. Then the concept “topological immunity” is applied to present the propagation of the flexural wave around the circular defect. This phenomenon makes the active controllable structure can immunize to the object, which means that the metamaterial plate exhibits a robust property at a certain frequency. Then, we can design an elastic wave metamaterial plate as a new function to achieve the topological immunity of the flexural wave. This work demonstrates the similar physics to ref. ^40^ for elastic wave and an additional tuning approach. Due to both the P-symmetry and T-symmetry can be found, the Berry curvature and Chern number are zero. The periodic structure is excited by a shaker at the right boundary and the frequency response is considered.

**Figure 3:**
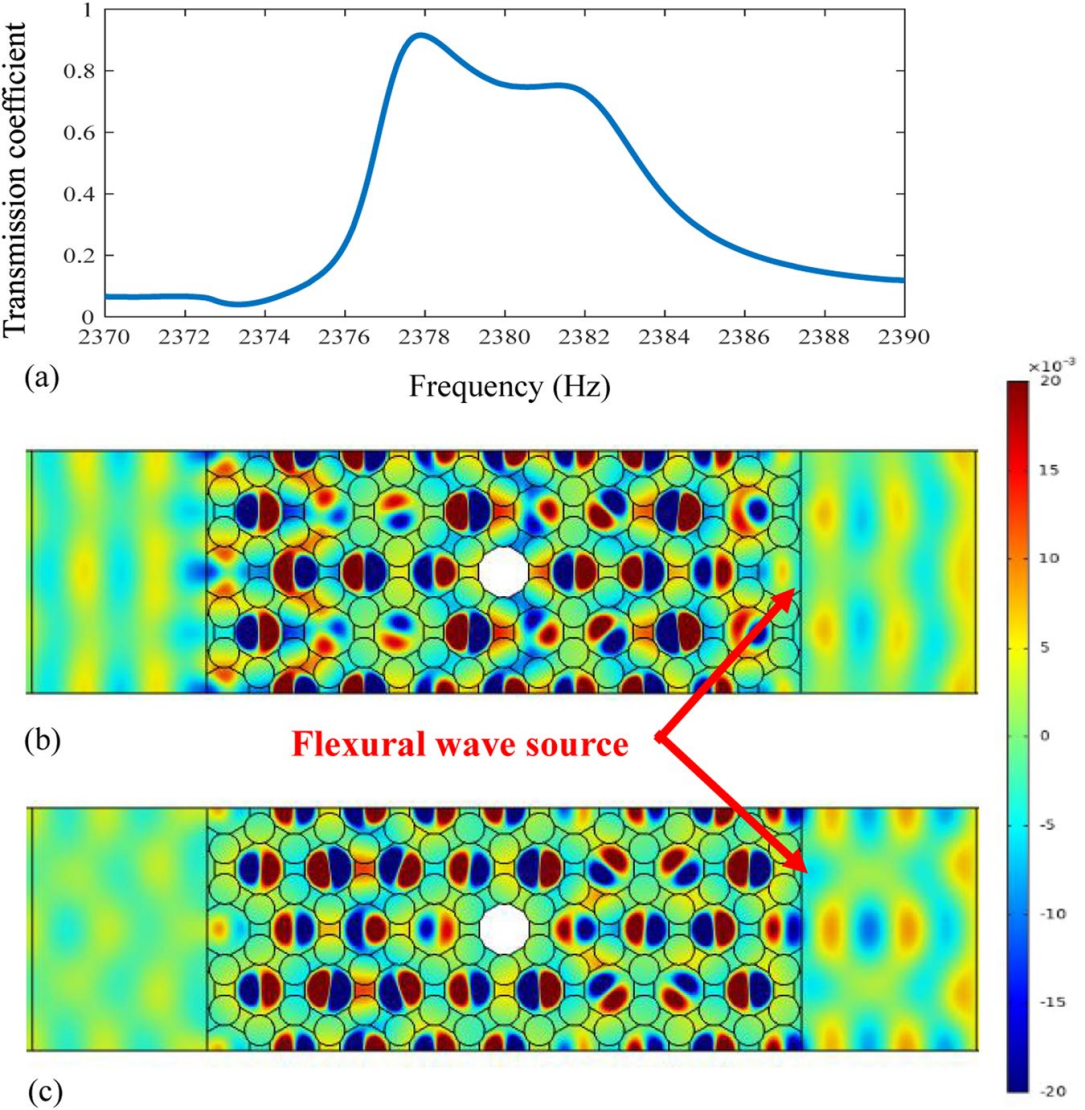
(**a**) Transmission coefficient, (**b**) wave propagation at 2377.9Hz with electrical circuits and (**c**) wave propagation without electrical circuits at 2377.9 Hz.

Here we define the transmission coefficient as the ratio of the left to the right flexural displacements. From the transmission coefficient curve for the elastic wave metamaterial plate with a defect in Fig. 3(a), a peak with 0.91 at the frequency of 2377.9 Hz is found. At this frequency, the flexural waves are almost perfectly transmitted through the whole system. Furthermore, when the frequency of the incident wave is 2377.9 Hz which is close to the Dirac point, the phase change becomes almost invisible in Fig. 3(b). Although there is a weak reflection on the right side, the defect is undetectable from the left field. Generally speaking, 2377 Hz does not shift obviously compared to 2256 Hz. It is mainly because of the problems during the mesh generation and boundary condition setting. An important influence is that the band structure is calculated with the infinite periodic boundary condition but the transmission spectrum is calculated with finite periodic structures.

In order to support these numerical results, the transmission response of the metamaterial plate without external circuits is calculated and shown in Fig. 3(c). We can see that the transmission becomes low and the waveform is observably distorted, which means that the topological immunity is achieved by the active control. The tunable effects of the active control on the topological immunity are considered, in which the negative capacitance circuits are applied to generate the Dirac point. When the Dirac point appears in the band structure, the topological immunity in the elastic wave metamaterial plate can be found. At the same time, there is only one peak in the transmission coefficient curve, which corresponds to the Dirac point.

Then, the active control experiments are performed on the elastic wave metamaterial plate in which the P-4 piezoelectric patches are boned at the corners. The elastic wave metamaterial plate is fabricated by the 3D printing technology with 8000 synthetic resin, in which the Young’s modulus *E* = 2.5 GPa, density *ρ* = 1300 kg/m^3^ and Poisson’s ratio *ν* = 0.41. The material parameters of the P-4 piezoelectric patch are shown in Table2 and the operational amplifier LM324N is applied.

**Table 2:** The material parameters of the piezoelectric patch.

Material	Mass density *ρ* (kg/m^3^)	Young’s modulus *E* (N/m^2^)	Compliance coefficient ${{s}}_{11}^{{E}}$ (m^3^N^−1^)	Piezoelectric strain coefficient *d*_31_ (Cm^−2^)	Permittivity ${\varepsilon }_{33}^{T}$ (Fm^−1^)
P-4	7450	8.83 × 10^10^	1.2 × 10^−11^	−1 × 10^−10^	−1 × 10^−10^

**Figure 4:**
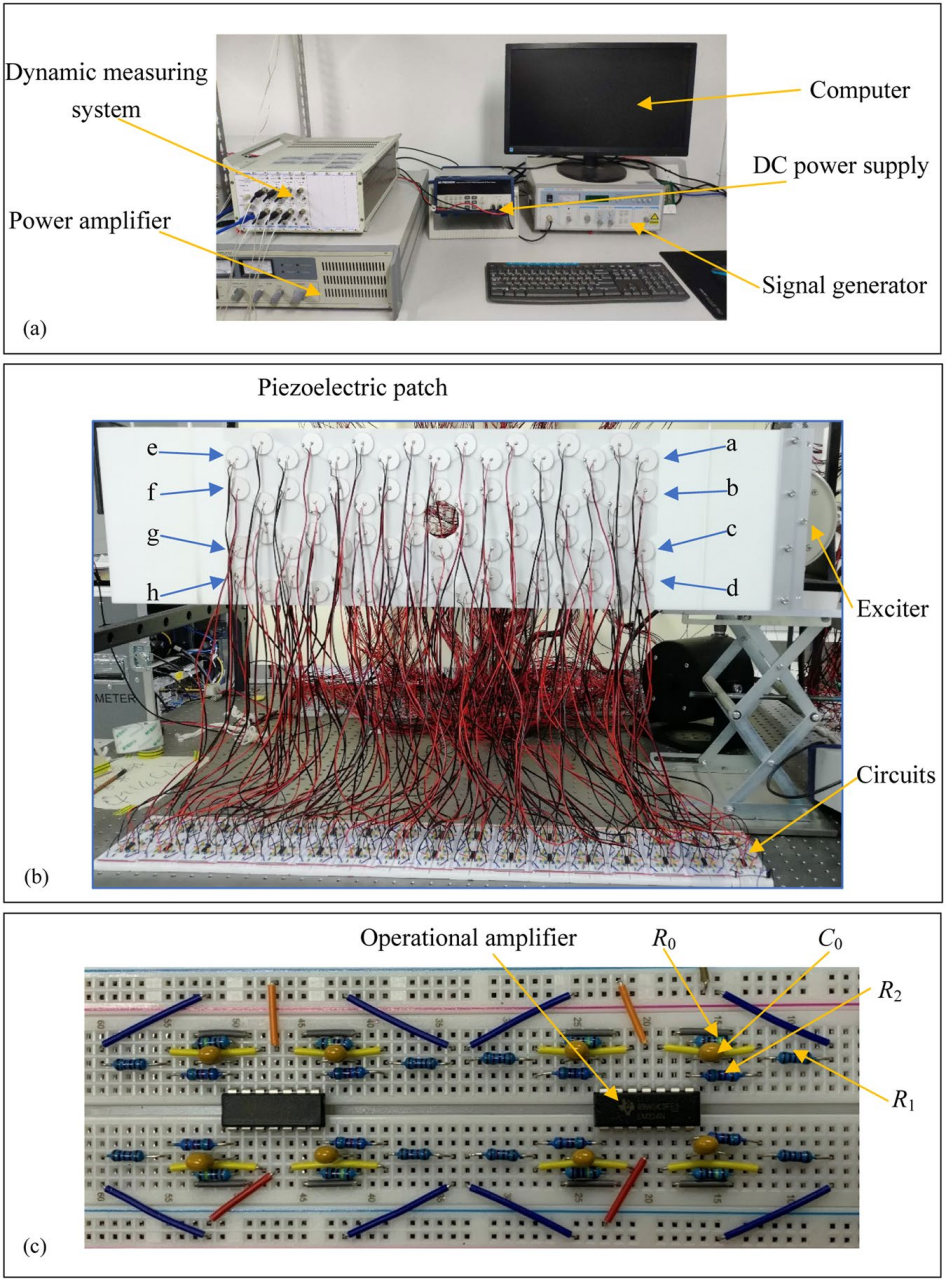
Experimental setup: (**a**) The testing system, (**b**) the whole structure with the active control system and (**c**) the negative capacitance circuit.

The experimental setup is illustrated in Fig. 4(a), in which the elastic wave metamaterial plate is connected by the negative capacitance circuits. The exciter and the four received points e–h are illustrated in Fig. 4(b). The plate and exciter are connected by a fixture with a high rigidity to generate the flexural wave. During the experiment, 4 × 9 unit cells are applied to the elastic wave metamaterial plate and the sample size in Fig. 4(b) is same as that in Fig. 3(b). On the other hand, the power supply depends on the operational amplifier which is shown in Fig. 4(c). The description of the circuit is listed in Table 3. Due to the limitation of experimental conditions, only the finite periodic structures can be used for to support the numerical simulation. Although there is a little difference between the experimental and the numerical results, similar topological immune phenomenon can also be found during the experiment.

**Table 3:** The parameters of the negative capacitance circuit.

*C* (pF)	*C*_*p*_ (pF)	*R*_2_ (kΩ)	*R*_1_ (kΩ)	*R*_0_ (kΩ)	Operational amplifier
9.684	8.155	51.54	68	2000	LM324N

**Figure 5:**
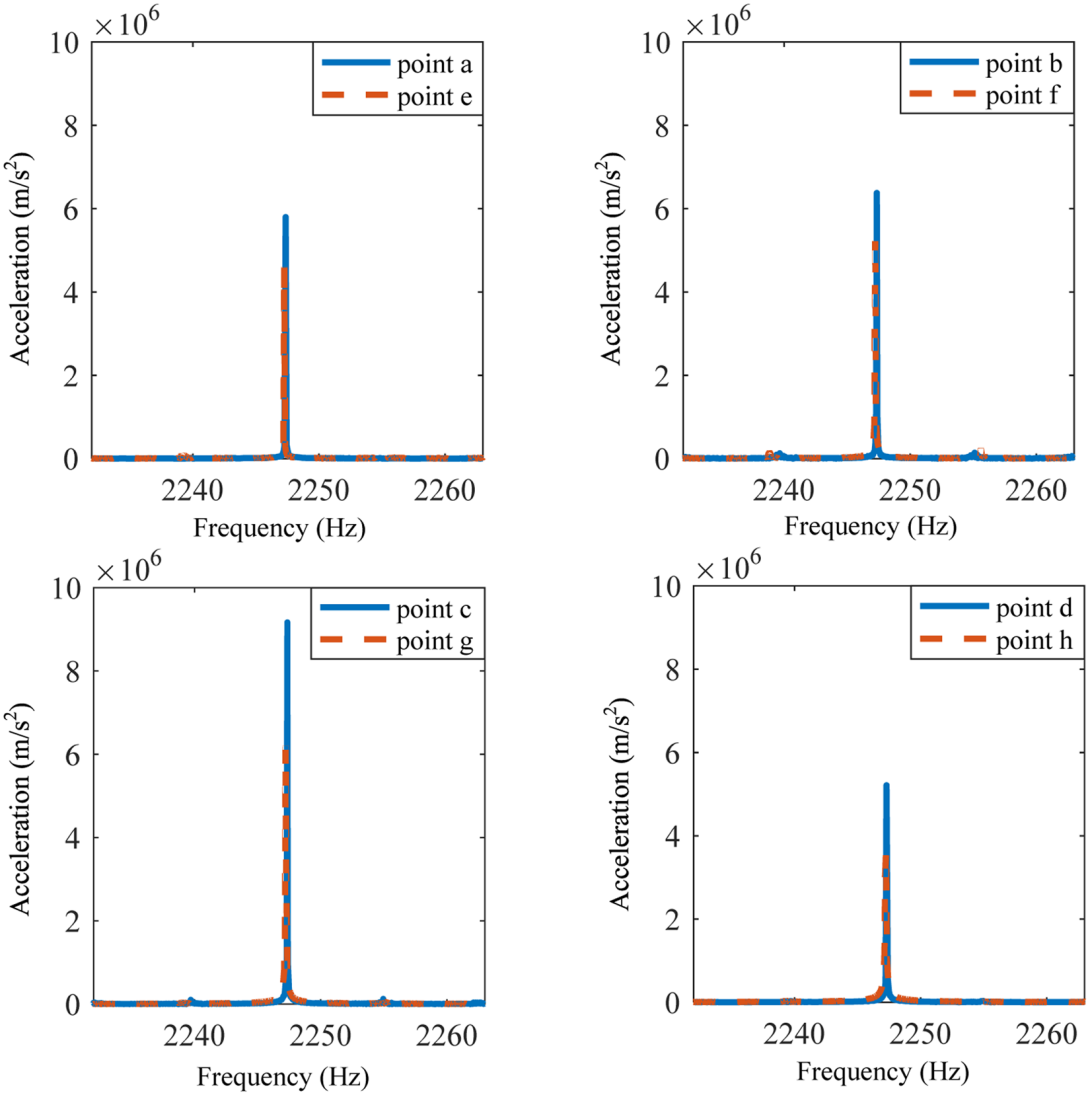
Responses of points a-h at 2247 Hz when the elastic wave metamaterial plate is attached by the active control system.

**Figure 6:**
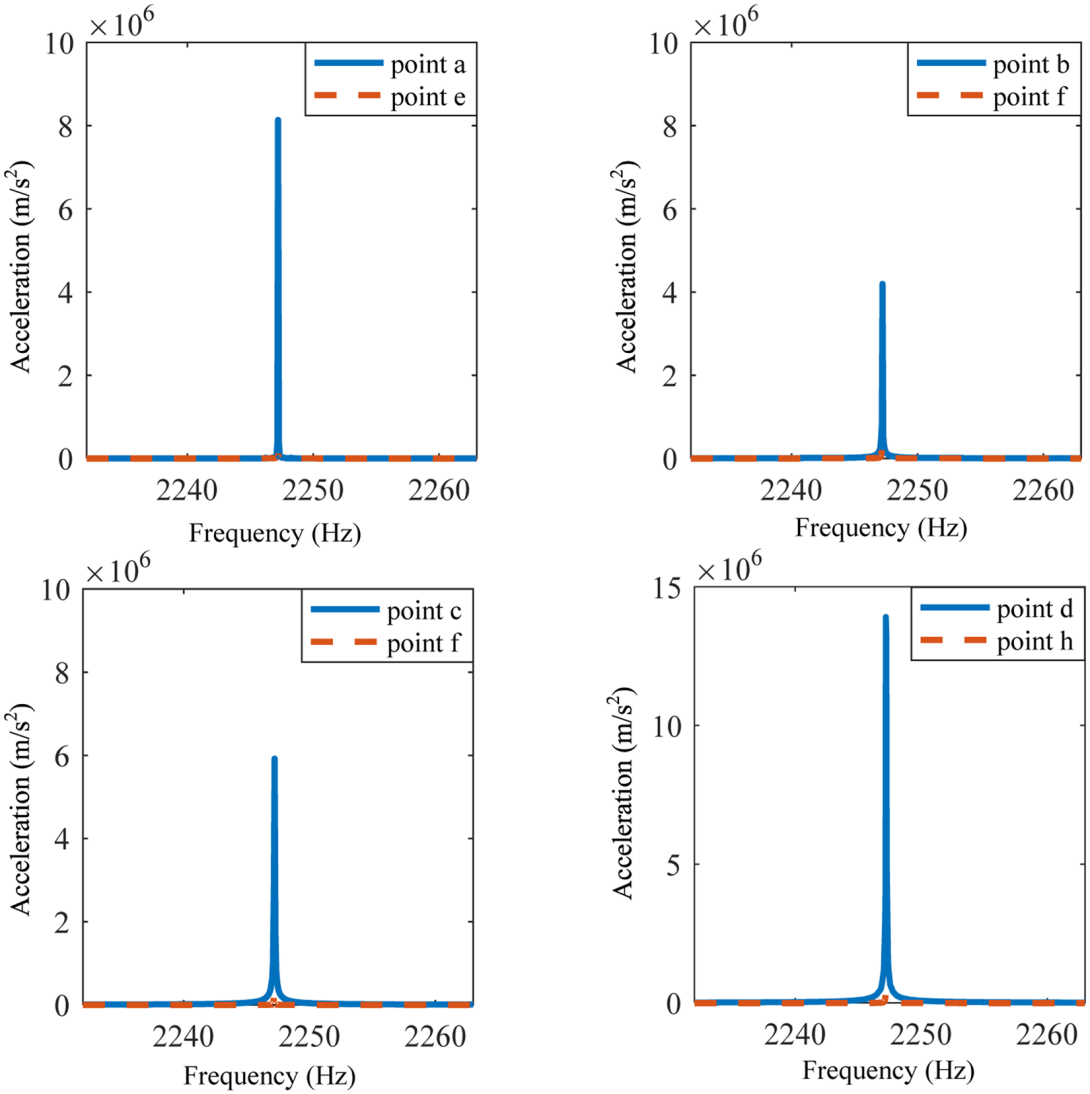
Responses of points a-h at frequency at 2247Hz when the elastic wave metamaterial plate is not attached by the active control system.

Figures 5 and 6 show the experimental results, in which the solid lines correspond to the points a–d (wave source) and dotted lines represent points e–h (response). With the connecting circuits on the elastic wave metamaterial plate in Fig. 5, we can see that although there is a little difference between the exciting and receiving points, their responses are quite similar at 2247 Hz. According to both numerical and experimental results, the waveform keeps the same from the right to the left sides. It means that the topological immunity is realized by the active control action. Then, the experiment is performed for the periodic structure without active control systems at the same frequency. As shown in Fig. 6, we can obviously see that the responses of the points e–h are quite smaller than those of the points a–d. It denotes that the flexural wave signal cannot be received on the left side for the structure without the active control.

## Conclusions

In this work, an elastic wave metamaterial plate with the active control system is design to illustrate the topological immune property. The band structure is calculated by the numerical simulation and the double Dirac point is achieved by the external negative capacitance circuits. Around the Dirac point, the transmission responses of the metamaterial plate with a defect are discussed. Experiments are performed with the 3D printing metamaterial plate being bonded by the external electrical circuits. The topological immunity is observed by the responses behind the defect and achieved by the control action.

## Supplementary information

Supplementary Information.
