# Supplementary material for: Active control on topological immunity of elastic wave metamaterials
Source: Sci Rep. 2020 Jun 10;10:9376. doi: 10.1038/s41598-020-66269-2 (PMC7287085; doi:10.1038/s41598-020-66269-2)
Supplement: Supplementary file 1 — Supplementary Information. [file 41598_2020_66269_MOESM1_ESM.doc]

**Supplementary Material**

**Active control on topological immunity of elastic wave metamaterials**

Guan-Hua Li ^1^, Tian-Xue Ma ^2^, Yi-Ze Wang 3 [[1]](#footnote-2)*, Yue-Sheng Wang ^1, 3^

^1^ Institute of Engineering Mechanics, Beijing Jiaotong University, Beijing 100044, China

^2^ Department of Civil Engineering, University of Siegen, Siegen, D-57068, Germany

^3^ Department of Mechanics, Tianjin University, Tianjin, 300350, China

The *x*, *y* and *z* directions are denoted as 1, 2 and 3, respectively. Assuming the polarization direction is along the *z*-axis, the constitutive equation of piezoelectric materials can be expressed as [1]

$\begin{matrix} S_{1}=s_{11}^{E}T_{1}+d_{31}E_{3} \\ D_{3}=d_{31}T_{1}+\varepsilon_{33}^{T}E_{3} \end{matrix}$, (S1)

where *S*_1_ and *T*_1_ are the strain and stress along the *x* direction; $s_{11}^{E}$ is the compliance coefficient; *d*_31_ and $\varepsilon_{33}^{T}$ are the piezoelectric and dielectric constants; *E*_3_ is the electric field intensity and *D*_3_ is the electric displacement along the *z-*direction.

Then, the relation between the strain and stress can be derived as [2]

$S_{1}=\left(s_{11}^{E}-\frac{sZd_{31}^{2}A_{s}h_{p}^{-1}}{1+sZC_{p}}\right)T_{1}$, (S2)

where *s* is the Laplacian parameter; *Z* is the complex impedance of the shunting circuit; $C_{p}=\varepsilon_{33}^{T}A_{s}\left(h_{p}\right)^{-1}$is the inherent capacitance of the piezoelectric patch; *A_s_* is the electrode area and *h_p_* is the patch thickness.

The inherent elastic modules of the patch can be derived from Eq. (S2) as

$E_{p}=\frac{h_{p}\left(1+sZC_{p}\right)}{h_{p}s_{11}^{E}\left(1+sZC_{p}\right)-sZd_{31}^{2}A_{s}}$. (S3)

And the complex impedance *Z* can be defined as [2, 3]

$Z=\frac{1}{-\alpha C_{p}s}$, (S4)

where *α*=(*R*_2_×*C*)/(*R*_1_×*C_p_*).

Therefore, we can tune *α* to change the elastic modules of piezoelectric patches, which can perform as the active control action on the elastic wave.

**References**

1. Hagood, N.W., Flotow, A.V., 1991. Damping of structural vibrations with piezoelectric materials and passive electrical networks. Journal of Sound and Vibration 146, 243–268.
2. Wang, G., Chen, S., 2015. Large low-frequency vibration attenuation induced by arrays of piezoelectric patches shunted with amplifier–resonator feedback circuits. Smart Materials and Structures 25, 015004.
3. Chen, S.B., Wen, J.H., Wang, G., Yu D.L., Wen, X.S., 2016. Directionality of wave propagation and attenuation in plates with resonant shunting arrays. Journal of Intelligent Material Systems and Structures 27, 28–38.

1. ^^ Corresponding author

   *Email addresses:* wangyize@tju.edu.cn (Y.-Z. Wang) [↑](#footnote-ref-2)
